# Bruns Syndrome is a Common Presentation of Neurocysticercosis

**DOI:** 10.4269/ajtmh.15-0374a

**Published:** 2015-09-02

**Authors:** A. Clinton White

**Affiliations:** Infectious Disease Division Department of Internal Medicine University of Texas Medical Branch Galveston, TX E-mail: acwhite@utmb.edu

Dear Sir:

Shahani, Garnes, and Mejia are to be congratulated on highlighting the importance of Bruns syndrome as a presentation of neurocysticercosis and the importance of neurosurgery for this condition.^1^ However, contrary to what is stated in their discussion, Bruns syndrome is a common presentation of neurocysticercosis that has been well recognized since Bruns' initial case report in 1902 of a patient with a fourth ventricular cysticercus.^2^ By the 1950s, Bruns syndrome caused by neurocysticercosis was widely recognized. Bickerstaff in 1955 felt the need to explain that there were other causes of the syndrome besides fourth ventricular cysticercosis.^3^ Bruns syndrome due to cysticercosis has also been frequently reported from the United States, including 11 cases described by McCormick in 1985.^4^ Indeed, symptoms of Bruns syndrome have been included in all case series of ventricular cysticercosis, including a number at Shahani and others' own institution.^5^–^7^ Rather than 14 cases, there are actually at least 100 cases in the literature.

Furthermore, as chair of the ASTMH/IDSA guidelines committee for management of neurocysticercosis, I mention some concerns about the management of the described cases. First, experts agree that, despite their widespread availability from commercial laboratories, enzyme-linked immunosorbent assays for antibody have little or no role in the diagnosis of neurocysticercosis. This conclusion is based on numerous studies demonstrating high rates of false positive and false negative serology. Second, the role of antiparasitic drugs in ventricular neurocysticercosis is controversial. In reviewing the literature, I could find no role for preoperative antiparasitic drugs in ventricular cysticercosis. Indeed, cysticidal drugs can make the cysts more friable, leading to poorer surgical outcomes. Furthermore, unlike hydatid disease, cysticerci only have a single scolex, and pose no risk for spread when ruptured. Although antiparasitic treatment is an important component of management of neurocysticercosis, it is never urgent. It should only follow and never precede management of hydrocephalus.^7^ Finally, the role of postoperative chemotherapy is also controversial. Many centers treating large numbers of cases do not give any chemotherapy after successful cyst removal and have not noted recurrences. The data supporting postoperative chemotherapy are mostly based on studies employing earlier generation neuroimaging, which is less sensitive than current methods.
